# The contribution of a MOET nucleus scheme for the improvement of Guzerá (*Bos indicus*) cattle for milk traits in Brazil

**DOI:** 10.3389/fgene.2022.982858

**Published:** 2022-09-29

**Authors:** Maria Gabriela Campolina Diniz Peixoto, Eula Regina Carrara, Paulo Sávio Lopes, Frank Ângelo Tomita Bruneli, Vânia Maldini Penna

**Affiliations:** ^1^ Embrapa Gado de Leite, Embrapa, Juiz de Fora, Minas Gerais, Brazil; ^2^ Departamento de Zootecnia, Universidade Federal de Viçosa, Viçosa, Minas Gerais, Brazil; ^3^ Centro Brasileiro de Melhoramento Genético do Guzerá, Belo Horizonte, Minas Gerais, Brazil

**Keywords:** selection, genetic progress, phenotypic progress, zebu cattle, dairy cattle

## Abstract

The Guzerá breed evolved from the introduction of breeds from India, mainly the Kankrej breed, into Brazilian livestock at the end of the 19th century. Guzerá adapted well to the climatic conditions of Brazil, where it is considered a dual-purpose breed and has been used for pasture-based beef, milk or dual-purpose production systems with the use of low-medium inputs. The importance of this genetic resource for milk production in tropical regions moved breeders to implement the National Breeding Program for the Improvement of Guzerá in 1994, based on both progeny testing and MOET nucleus schemes. We sought to evaluate the role of the MOET nucleus scheme in the phenotypic and genetic progress for milk traits in this breed. The initial database used in the present study consisted of 6,513 cows, daughters of 761 bulls. We performed genetic evaluations with different datasets using a linear mixed model in a single trait analysis, including the relationship matrix, in order to estimate breeding values. Inbreeding coefficients were also calculated using the relationship of descent between two parents. Annual phenotypic, genetic and inbreeding trends were obtained for each dataset, considering the genetic pathways of both the bull and the cow. The low genetic progress found for milk yield in the whole population (5.27 ± 0.30 kg/year) partially accounted for the dual-purpose selection goal, despite the higher genetic progress in the MOET nucleus (9.39 ± 0.79 kg/year). The inbreeding coefficient was minimized at the beginning of the breeding program based on the use of new lineages. Posteriorly, it started increasing again from 0.002 in 1991 to 0.008 in 2019. The results provided evidence of the significant contribution of the MOET nucleus scheme for the phenotypic and genetic progress of Guzerá breed for milk traits, as well as of the impact of the breeding program on the inbreeding coefficient rate in the early years. New strategies need to be designed for the Guzerá breed, to allow for greater improvement of milk traits and minimizing the rate of the inbreeding coefficient.

## 1 Introduction

Nucleus-breeding schemes present an interesting approach to dairy cattle improvement. These schemes may be effective in organizing the breeding program, particularly when large-scale recording is lacking, for comparing animals under standard conditions (Hinks, 1977; Smith, 1988). Many authors highlight advantages of these schemes, among them, the possibility of an initial genetic lift (McDaniel and Cassel, 1981; Nicholas and Smith, 1983; Teepker and Keller, 1989). However, it is important to have a large base population, and to minimize the harmful effects of inbreeding and genetic drift (Nicholas and Smith, 1983; Juga and Mäki-Tanila, 1987; Smith, 1988; Woolliams, 1989; Ruane, 1991; Ruane and Thompson, 1991).

Multiple ovulation and embryo transfer (MOET) allow an increase in family sizes, mainly in collateral relatives, enabling improvement of the nucleus-breeding scheme (Nicholas and Smith, 1983; Colleau and Mocquot, 1989; McGuirk, 1989; Teepker and Keller, 1989; Woolliams and Wilmut, 1989). The use of embryo transfer allowed obtainment of groups of full-sisters and half-sisters, for a pre-assessment of young bulls entering the progeny test (PT) (Smith and Ruane, 1987). However, while in the PT-based schemes the selection of males is more intense than of females, in the nucleus, the intensity of the selection of the females’ pathway could be greater, due to the increase in the number of progeny per cow (Land and Hill, 1975).

The use of MOET also allowed reduction of the generation interval, by making it possible for the mothers of the bulls to produce a greater number of progeny in a shorter period than it would normally take during the animal’s life (McDaniel and Cassel, 1981). Furthermore, greater accuracy and selection intensity in PT-based schemes implies greater reduction in genetic variance. Thus, the long-term genetic progress possible in the nucleus breeding schemes could even be underestimated, and could be higher than that based on the PT (Nicholas, 1979; Meuwissen, 1991a). Therefore, decreases of the generation interval would imply greater genetic progress.

Factors of genetic progress variation in nucleus schemes such as herd size, mating system, sibling family type, selection age, number of sires selected per full-sib group, have also been studied (Nicholas and Smith, 1983; Juga and Mäki-Tanila, 1987; Ruane and Thompson, 1991). Nicholas and Smith (1983) suggested the selection of only one male per full sib group to minimize the inbreeding rate. However, this can reduce the genetic progress by not exploring variation within the family (Ruane, 1991). Woolliams (1989), on the other hand, suggested the random selection of males from a greater number of full-sib families, thereby increasing selection pressure and minimizing inbreeding.

Some authors have proposed a nucleus that is continuously open to the introduction of individuals from other herds of known genetic merit (Juga and Mäki-Tanila, 1987; Smith, 1988; Ruane and Smith, 1989; Meuwissen, 1991b; Bondoc and Smith, 1993). This would help to reduce levels of inbreeding and losses of genetic variance, as well as increase the rate of genetic progress, compared to closed schemes. Two other strategies focused on shortening the generation interval to 1.8 years and 3.7 years in the juvenile and adult designs, respectively (Nicholas and Smith, 1983), since the generation interval is one of the determining factors of annual genetic progress.

Zebu dairy herds in Brazil have a long generation interval as a result of both a late age at first calving and a large calving interval related to genetic and environmental circumstances (Nogueira, 2004), limiting the genetic progress rate. While in conventional PT of Zebu dairy cattle the generation interval under tropical conditions is around 6 years, in the nucleus scheme the animals would be selected earlier resulting in greater genetic progress. This outcome may even compensate for the lower accuracy of this scheme, and outperform the progress based on the progeny test scheme (Nicholas and Smith, 1983; Juga and Mäki-Tanila, 1987; Meuwissen, 1991b; Ruane, 1991).

Tropical dairy systems demand specific selection objectives and management geared to regional reality, focusing on adaption to the harsh environment, in order to become sustainable (Madalena et al., 2012). Regarding the environmental, economic and socio-cultural diversity of dairy in Brazil, understanding productive context is fundamental for designing breeding programs. Guzerá cattle is an important genetic resource for tropical production systems, and has been largely used in pure or crossbred herds, prevailing in Brazilian like-Savanah and semiarid regions. Due to its wide adaptation to environments and low quality of feed, parasite resistance, and dual-purpose characteristics, it has been selected for beef, dairy or both purposes in the country (Peixoto et al., 2021).

In 1994, the Brazilian Center for the Genetic Improvement of Guzerá (CBMG^2^), in partnership with Embrapa-Dairy Cattle, implemented the National Program for the Improvement of Guzerá for Dairy Purposes (PNMGuL). This program is based on both the progeny test and MOET nucleus schemes (Penna et al., 2002; Bruneli et al., 2021). The joint selection strategy aims at promoting an initial genetic lift and rapid genetic progress in milk production traits, using accurate breeding values (EBVs). Therefore, the objective of the present study was to evaluate the role of the adult open MOET nucleus in the results achieved in the PNMGuL.

## 2 Materials and methods

### 2.1 The multiple ovulation and embryo transfer nucleus scheme

The MOET nucleus scheme was initially financed by breeders and had the technical support of investigators of the Federal University of Minas Gerais and the Embrapa-Dairy Cattle Federal Research Organization. Many breeders comprised the effort to generate the families and bulls to be tested. The CBMG^2^ was responsible for the operational aspects. The MOET Nucleus sought to replicate the prevailing conditions of the Brazilian milk production systems based on Zebu animals in a tropical environment. That is, systems based on pasture with low-medium use of inputs, concentrated diet only for lactating cows, milking usually in the presence of the calf, and roughage supplementation during the dry period.

The MOET nucleus selection scheme of the Guzerá breed was based on the Taboquinha Farm, opened by Sinval Martins de Mello and Sons, in the municipality of Itambacuri, western Minas Gerais, Brazil. This municipality is in the Atlantic Forest biome, in the Rio Doce Valley, 334 m above sea level at the following geographic coordinates: latitude, 18° 2′ 40″ south and longitude, 41° 39’ 41” west. The climatologic averages observed from 30°years of data reveals: temperatures between 15 and 32°C throughout the year, rarely under 12°C in the dry period (April–September) or above 37°C in the rainy period (October to March); precipitation between 20 and 209 mm; and, humidity varying between 1 and 86%.

MOET was conducted in a commercial corporation. Following the development of *in vitro* embryo production biotechnology (IVP), the MOET biotechnology was replaced and IVP became the technique of choice for embryo generation, mainly after the success of *in vitro* fertilization (IVF) in Zebu breeds. However, the original denomination MOET Nucleus was kept.

Initially, to constitute the families in each annual battery, bulls and cows with the potential to maximize milk production were chosen based only on their phenotypes for milk and beef production. These phenotypes comprised the following data: lactation records from ancestors, collaterals, descendants and, in the case of cows, the lactation record itself. Subsequently, in the posterior batteries, the choice of parents for the next generation (later batteries) was based mainly on the following criteria: EBVs from the most recent genetic evaluation as well as on genealogy, inbreeding coefficient, and average relationship. Since the beginning, a commission formed by breeders and the Embrapa research team annually evaluates the proposed progeny test (PT) bulls and MOET families, and ranks those to be included in subsequent batteries.

Selected donors were sent for assisted reproduction at a private center. There, after an adaptation period, they were synchronized and super-ovulated. Embryos of satisfactory quality were then transferred to recipients (Holstein-Zebu crossbreeds). Initially, after the 2-months positive pregnancy diagnosis, these recipients were sent to the MOET Nucleus (Fazenda Taboquinha), 400 km distant from the embryo-transfer center, to give birth and where calves were raised. Males were sent to the MOET family’s owner after 15 months. Females were inseminated and kept at the Nucleus until the end of their first lactation. Subsequently, there was a change in the logistics at the Nucleus. The recipients started to be sent to the farms of the MOET family’s owners, and the calves were sent to the Nucleus after weaning. There, they were inseminated and kept until the end of their first lactation.

Milk production recording and individual milk sampling were performed monthly at the MOET Nucleus and in the PT herds. Individual milk samples were collected after each milking during the test-day using tubes containing Bronopol (Pestanol^®^, Sigma-Aldrich, United States). These samples were immediately refrigerated and sent to the Milk Analysis Laboratory at Embrapa Dairy Cattle for milk composition analysis. Data from test-day and milk composition were sent to the database team for computational processing. After processing, all these data were analyzed together with data from database of the Brazilian Association of Zebu Cattle. Joint genetic evaluations are carried out using the animal model methodology for BLUP evaluation, connecting data from all those sources. This procedure makes a significant contribution to the accuracy of breeding value for the animals born at the MOET Nucleus. Some proven bulls from this scheme were submitted to the progeny test in order to obtain additional accuracy. In sire summary, only bulls with minimum accuracy of 0.50 are listed, and few bulls have stayed below 0.60 in the last summary.

In response to concerns about the genetic variability and inbreeding coefficient, in 2009 animals from different origins than those used in the Nucleus, or those without milk production information, were used to produce MOET Nucleus families. The aim was to make available to the Guzerá herds bulls of alternative bloodlines with estimated genetic merit. These animals represented lineage or risk options and their origin and productive potential are, therefore, rare to the program. The number of risk animals included at the Nucleus is small and mating between them does not occur, avoiding the formation of “double risk” families. It was recommended that risk bulls were mated with “safe” cows and vice-versa.

### 2.2 Direction of parental choices

The number of cows (donors) and bulls for forming MOET families at each battery were variable, depending on the logistic and financial circumstances, oscillating from 4 to 16 donors and 4–13 bulls at each battery. Eventually, some bulls were mated to more than one cow.

#### 2.2.1 Cows (donors)

1- Cows to maximize milk (minimum 1/3 of the vacancies)—cows with PTA (predicted transmitting ability) > 300, choosing among them, preferentially, the 1st within each lineage, followed by those with the highest number of ancestral selection and lastly, those with good conformation and type. If there are no candidates with a PTA >300 available, use the ones with the highest PTA.
2- Lineage option cows—low risk—cows with PTA >100 were chosen, prioritizing rarer genetics within the program, followed by milk PTA cows with good conformation and type.
3- Risk cows (maximum 1/3 of the vacancies)—cows with innovative, and other, genetic attributes that are important for the program, as determined by the technical committee and breeders. Except in exceptional cases, the cow must have high production measured in a lactation.

#### 2.2.2 Bulls

1- Bulls to maximize milk (minimum half of the vacancies)—bulls chosen from the highest PTA, not yet used in the program, preferably one within each lineage (maximum one within each MOET family), with the highest number of generations of selection, and of better conformation and type of breed.
2- Bulls lineage and/or risk option (maximum half of the vacancies)—bulls prioritized for keeping the inbreeding coefficient (inbreeding) at minimum levels and adding favorable aspects in other traits, such as those morphological and growth. Chosen for new genetics or distant parentage, based on the best dairy information available. They must have breed and/or conformational attributes that are important to the breed.

### 2.3 Statistical analyses

#### 2.3.1 Data and model

Data for the present study were obtained from the National Program for the Improvement of Guzerá Cattle (PNMGuL) database. The complete dataset, including data from MOET Nucleus, was formed: from 6,513 first-lactation records from Guzerá cows, daughters of 761 bulls; the reduced dataset (without MOET Nucleus animals) from 4,482 Guzerá cows; and, 2,031 cows came from MOET Nucleus truncated to 305-days. Therefore, the traits evaluated were the 305-days first-lactation cumulative yields (kg) of milk (MY305), fat (FY305), and protein (PY305). The number of test-day records used for obtaining the cumulative yields were 7.6 ± 1.9 (milk yield), 5.8 ± 2.1 (fat yield), and 5.6 ± 1.9 (protein yield).

The complete relationship matrix comprised data from 120,655 animals. The reduced relationship matrix contained 75,378 animals (45,277 related to the MOET Nucleus), being 2,144 sires and 18,560 dams from 13 overlapping generations. The MOET animals came from 182 full sib families formed over the years.

The genetic evaluation was performed using the complete data and the reduced data. The following mixed linear model single trait was assumed:

y=Xb+Za+e

where y is the vector of phenotypes; b is the vector of fixed effects of a contemporary group and covariate; a is the vector of random additive direct genetic effects, with $a∼N\left(0,A\sigma_{e}^{2}\right)$ , where $\sigma_{e}^{2}$ is the additive direct genetic variance; A is the numerator relationship matrix; X and Z are incidence matrices related to the **b** and **a** to y , respectively; e is the residual vector, with $e∼N\left(0,I\sigma_{e}^{2}\right)$ , where $\sigma_{e}^{2}$ is the residual variance; and, I is an identity matrix of order equal to the number of animals.

Contemporary groups (CGs) were formed by combining herd, year, and season of calving (defined as dry [April–September] or rainy [October to March]). There were 58, 24, and 16 herds for respectively MY305, FY305 and PY305, and 604, 212, and 123 CGs were formed for MY305, FY305, and PY305. Data that belonged to the CG with fewer than three records and with only one sire as a parent were excluded. The age at calving was considered a linear covariate.

The genetic evaluation was performed using the complete data and the reduced data using BLUPF90 family programs (Misztal et al., 2014).

#### 2.3.2 Inbreeding coefficients

Inbreeding coefficients (F) were calculated using the relationship of descent between two parents X and Y; that is, the kinship coefficient ( $F_{XY}$ ), given by:

$$F_{xy}=\frac{1}{4\left(F_{AB}+F_{AD}+F_{BC}+F_{BD}\right)}$$

where A and B are parents of X, and C and D are parents of Y. The inbreeding coefficient of animal Z ( $F_{Z}$ ) is equal to the kinship coefficient of their parents X and Y; that is $F_{Z}=F_{xy}$ . This step was performed using the “Optimum Contribution Selection and Population Genetics” (optiSel) package (Wellmann, 2018) from R software (R Core Team, 2022).

Phenotypic and genetic trends for milk traits were estimated from the averages of the records and estimated breeding values (EBV) by birth year, considering the different datasets. In the same way, the averages of the inbreeding coefficients were regressed by birth year. Furthermore, EBV averages for cows and bulls were regressed separately by birth year, in order to evaluate the genetic contribution of each pathway for the improvement of the Guzerá breed for milk traits. In all cases, linear regressions were performed.

## 3 Results

The descriptive statistics are shown in Table 1. It is notable that around 30% of the cows with records came from the MOET Nucleus. Cows from MOET nucleus showed slight phenotypic superiority in milk and fat production.

**TABLE 1:** Number of animals with records (N), means and standard deviations (SD), and minimums (Min) and maximums (Max) of milk production traits in the Guzerá cattle database.

Phenotypes	N	Mean	SD	Min	Max
MY305 (complete data)	6,513	2,020.27	988.64	105.00	6,487.00
MY305 (reduced data)	4,482	1,990.81	973.69	105.00	6,163.00
MY305 (only data from MOET)	2,031	2085.28	1018.08	115.00	6,487.00
FY305 (complete data)	2,381	84.96	38.42	6.00	281.17
FY305 (reduced data)	1,433	91.03	40.64	6.00	281.17
FY305 (only data from MOET)	948	75.80	32.77	9.00	207.00
PY305 (complete data)	1,821	61.71	28.91	4.00	242.00
PY305 (reduced data)	828	62.87	31.36	4.00	242.00
PY305 (only data from MOET)	993	60.75	26.67	5.00	154.00

The annual phenotypic and genetic trends for MY305 considering complete data, reduced data and only data from the MOET Nucleus are shown in Figures 1–3, respectively, for all animals, and separately for males and females. Analogously, for FY305 and PY305, the annual genetic trends are shown in Figures 4–6 and in Figures 7–9, respectively.

**FIGURE 1:**
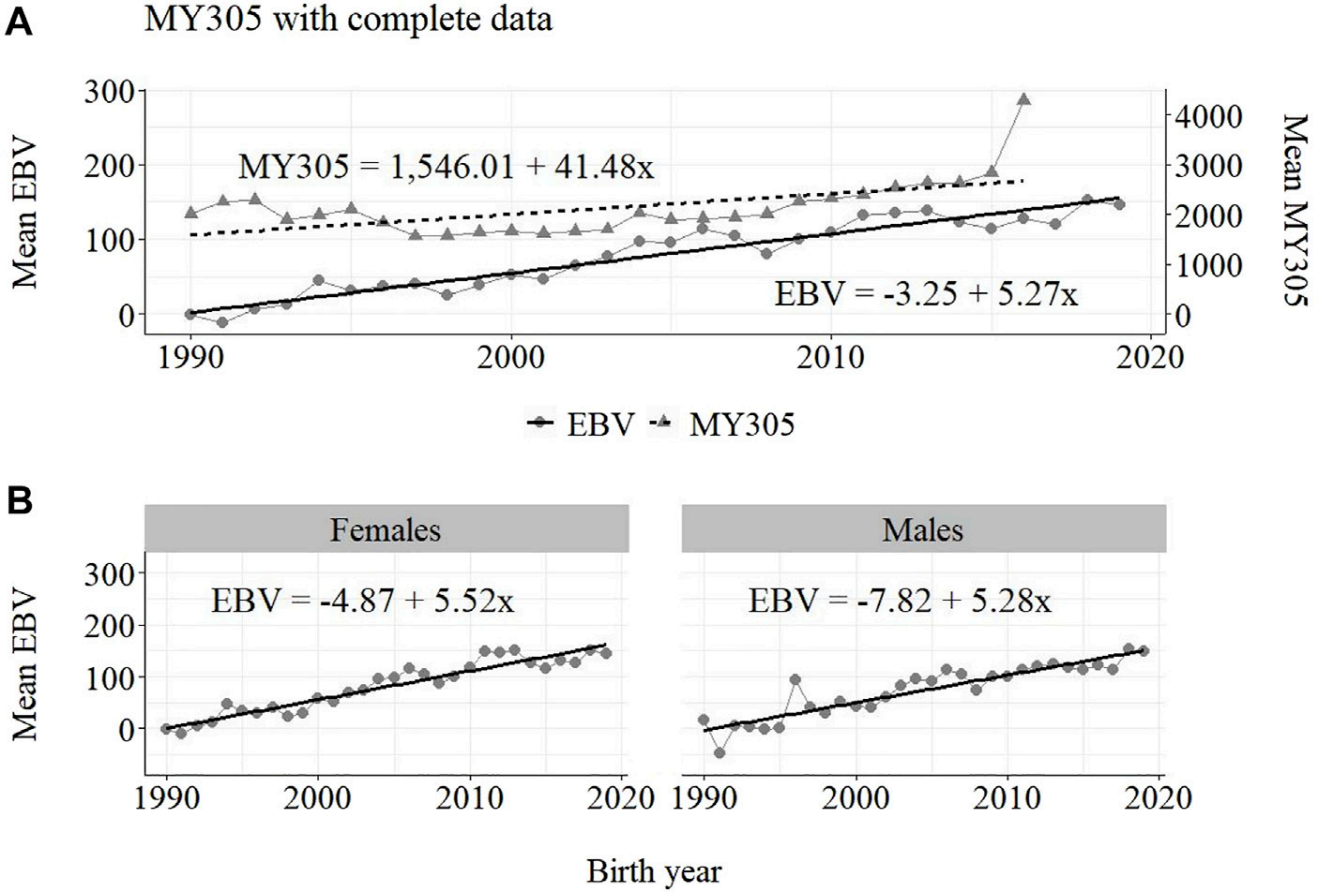
Annual phenotypic and genetic trends for MY305 of Guzerá animals for **(A)** all animals, and genetic trends for **(B)** males and females, considering data from the historical database, progeny test, and the MOET nucleus.

**FIGURE 2:**
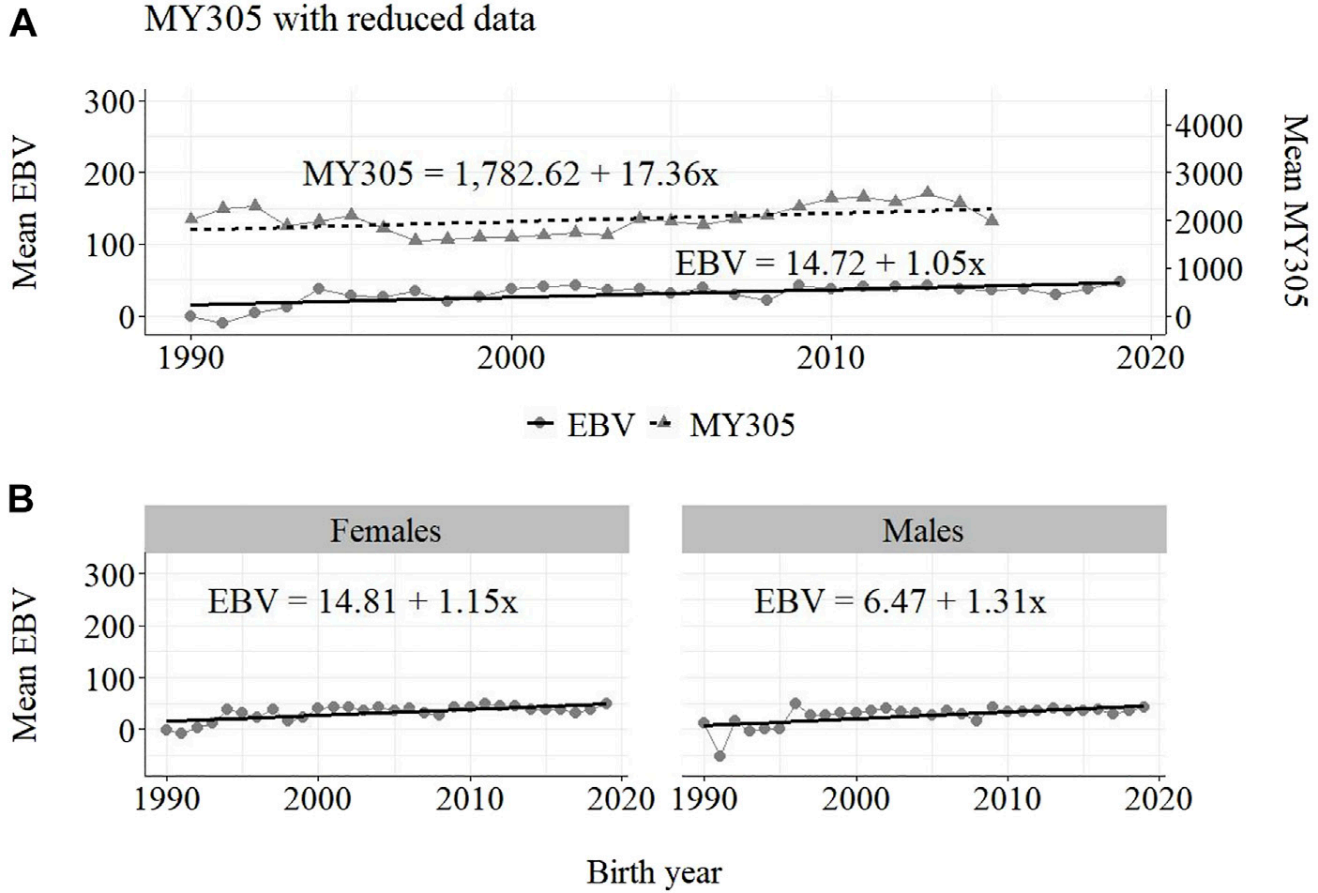
Annual phenotypic and genetic trends for MY305 of Guzerá animals for **(A)** all animals, and genetic trends for **(B)** males and females, considering only data from the historical database and progeny test scheme.

**FIGURE 3:**
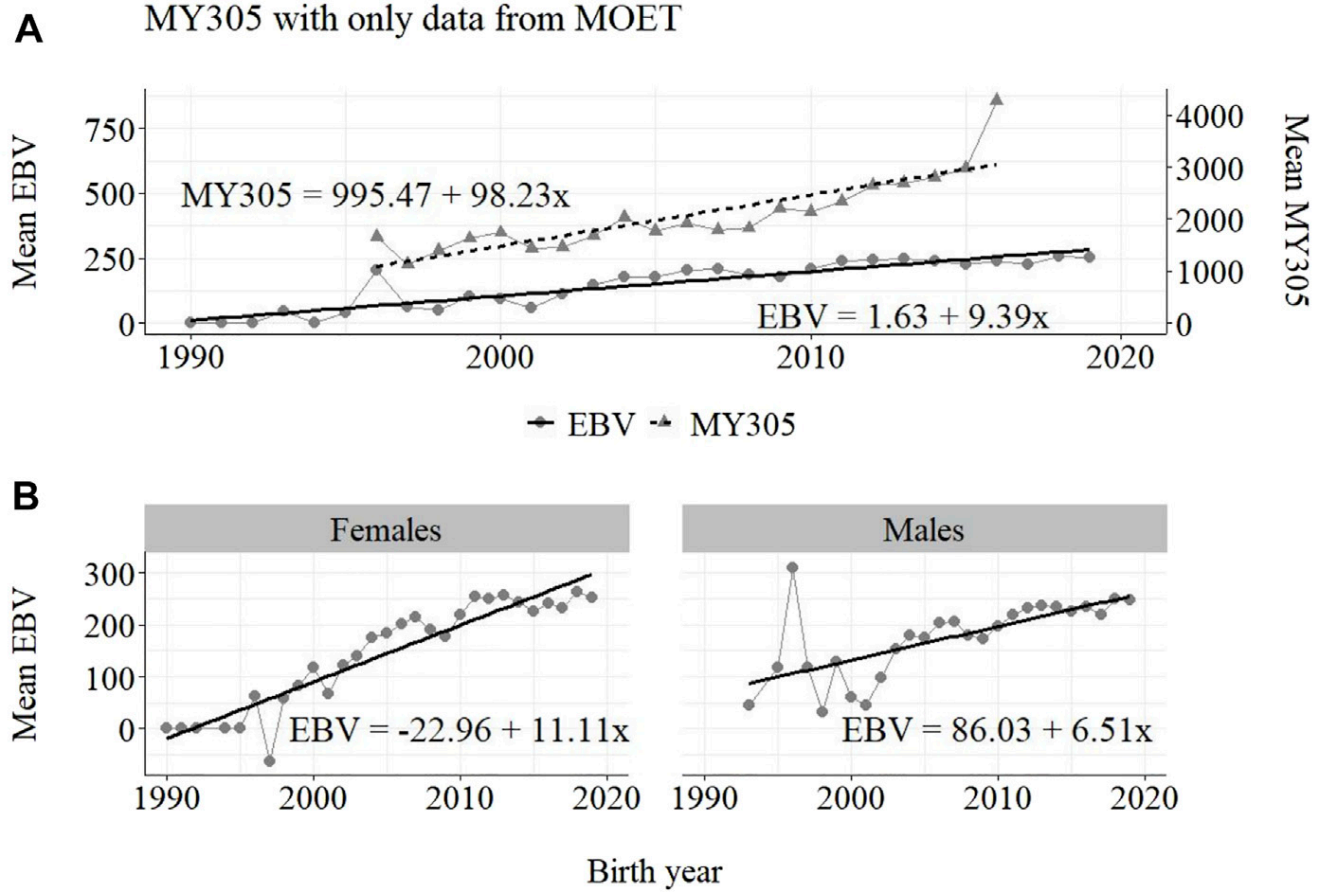
Annual phenotypic and genetic trends for MY305 of Guzerá animals for **(A)** all animals, and genetic trends for **(B)** males and females, considering only data from the MOET nucleus scheme.

**FIGURE 4:**
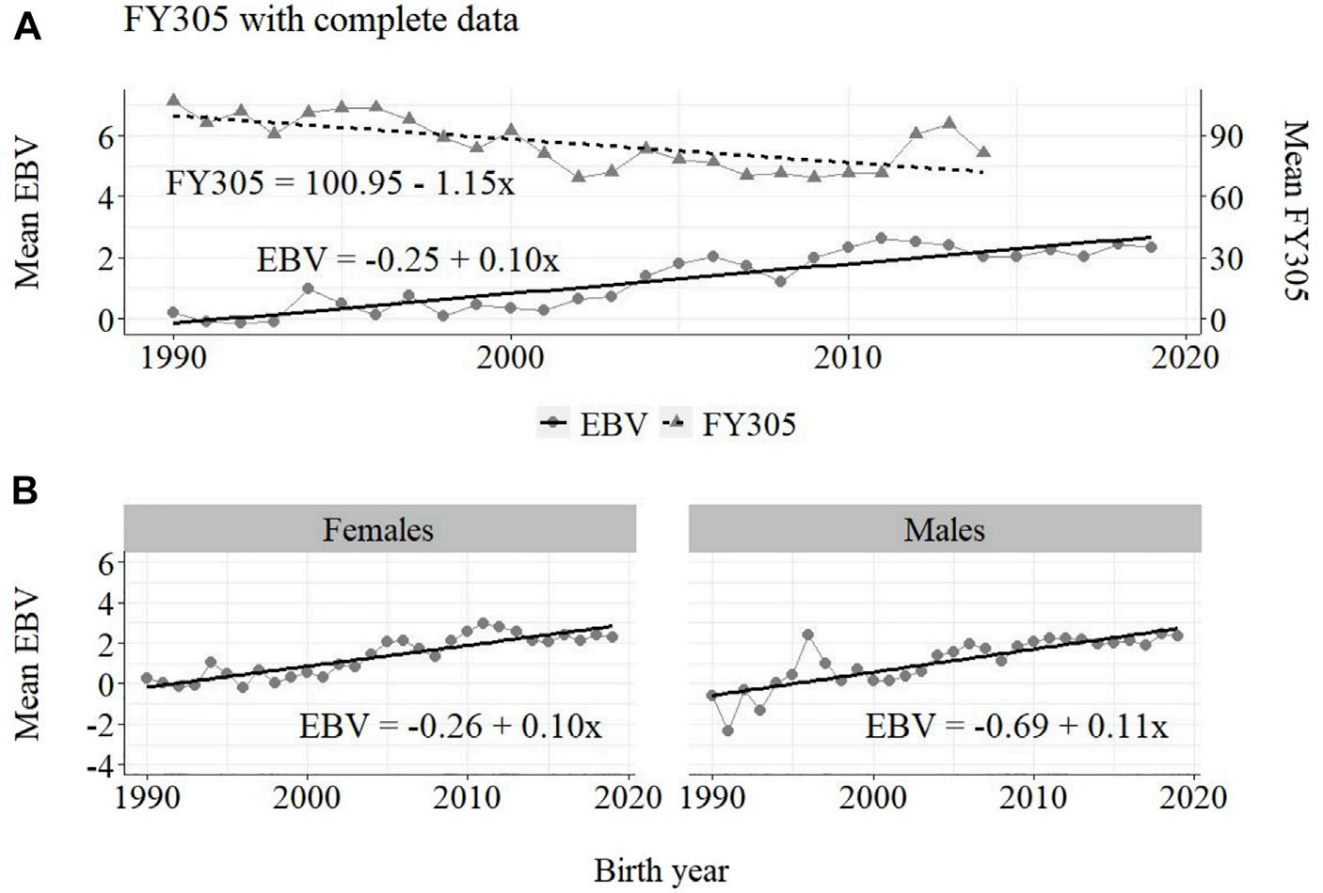
Annual phenotypic and genetic trends for FY305 of Guzerá animals for **(A)** all animals, and genetic trends for **(B)** males and females, considering data from the historical database, progeny test, and the MOET nucleus scheme.

**FIGURE 5:**
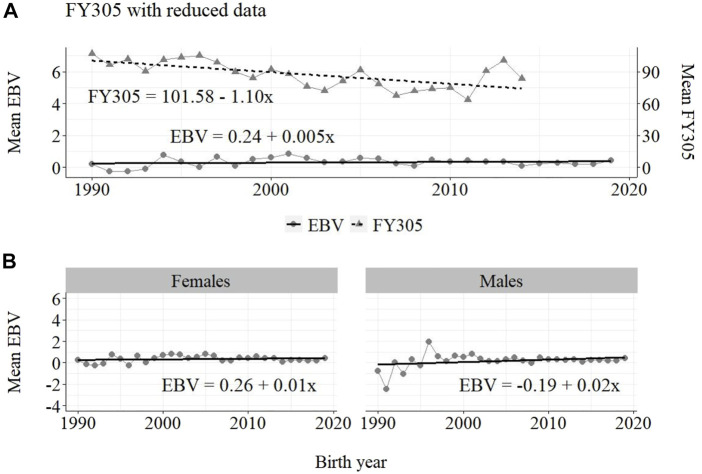
Annual phenotypic and genetic trends for FY305 of Guzerá animals for **(A)** all animals, and genetic trends for **(B)** males and females, considering data from the historical database and the progeny test scheme.

**FIGURE 6:**
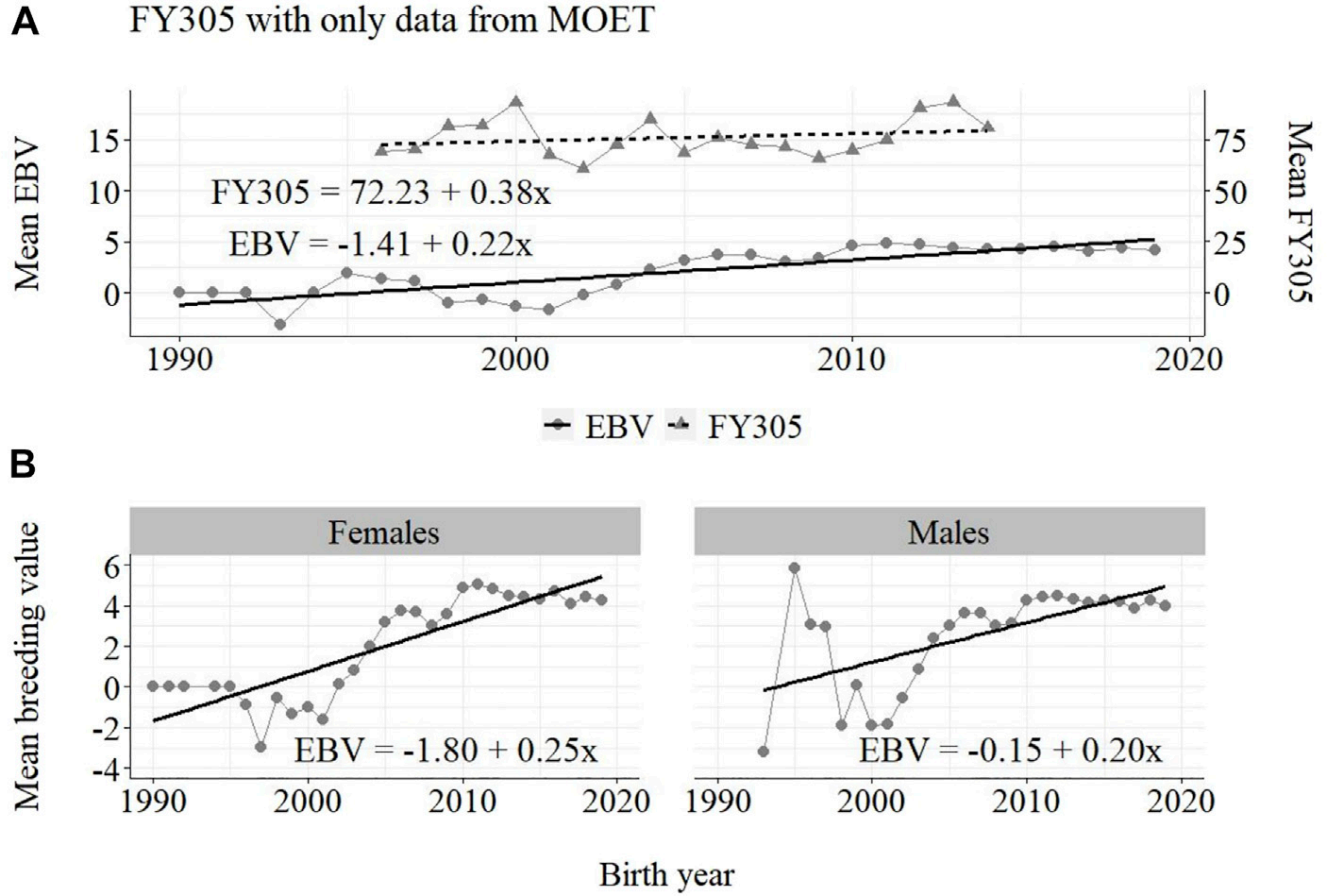
Annual phenotypic and genetic trends for FY305 of Guzerá animals for **(A)** all animals, and genetic trends for **(B)** males and females, considering only data from the MOET nucleus scheme.

**FIGURE 7:**
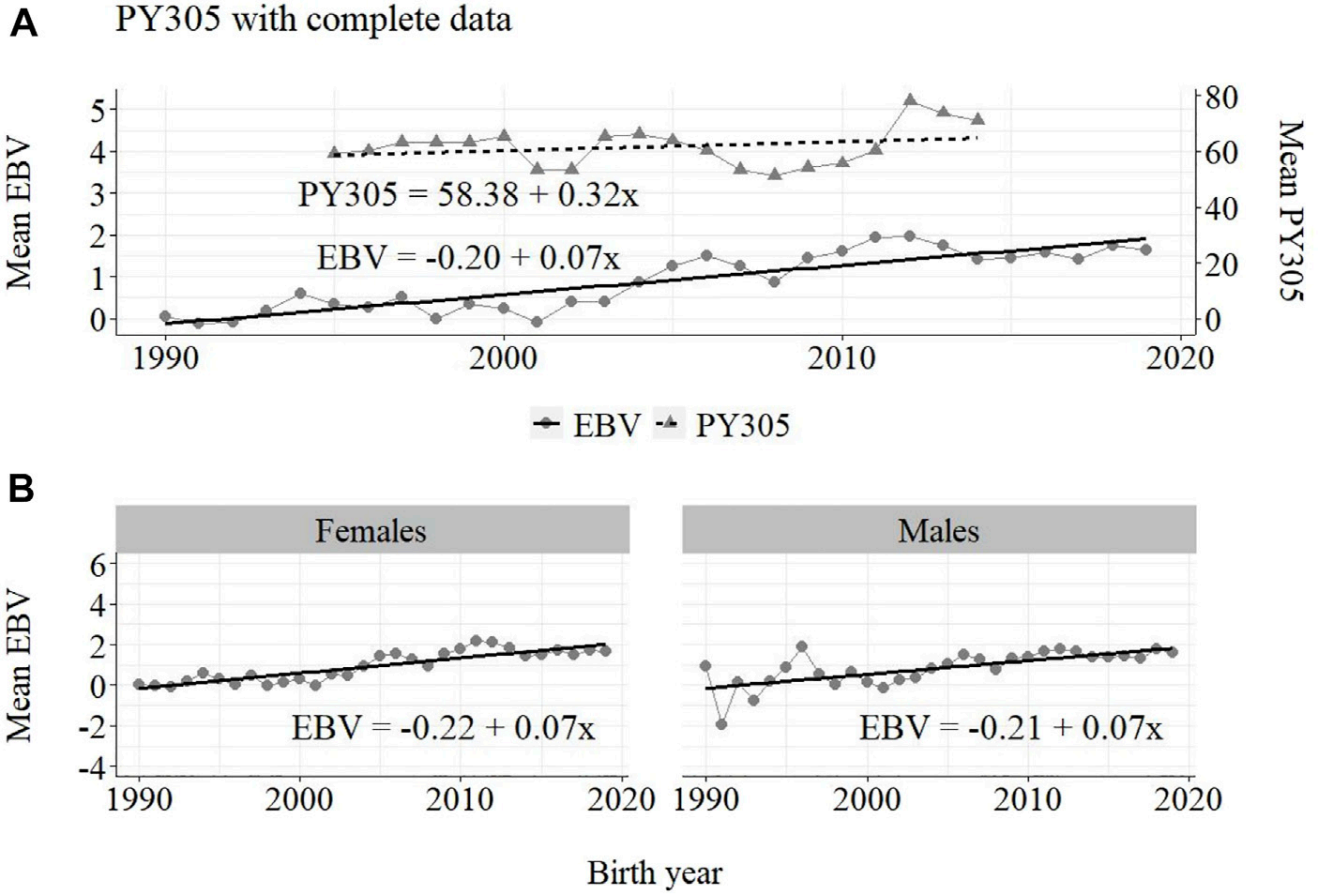
Annual phenotypic and genetic trends for PY305 of Guzerá animals for **(A)** all animals, and genetic trends for **(B)** males and females, considering data from the historical database, progeny test, and the MOET nucleus scheme.

**FIGURE 8:**
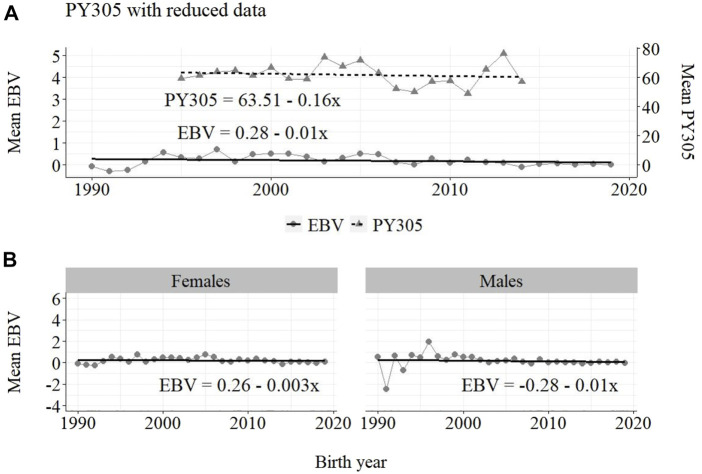
Annual phenotypic and genetic trends for FY305 of Guzerá females for **(A)** all animals, and genetic trends for **(B)** males and females, considering only data from the historical database and progeny test scheme.

**FIGURE 9:**
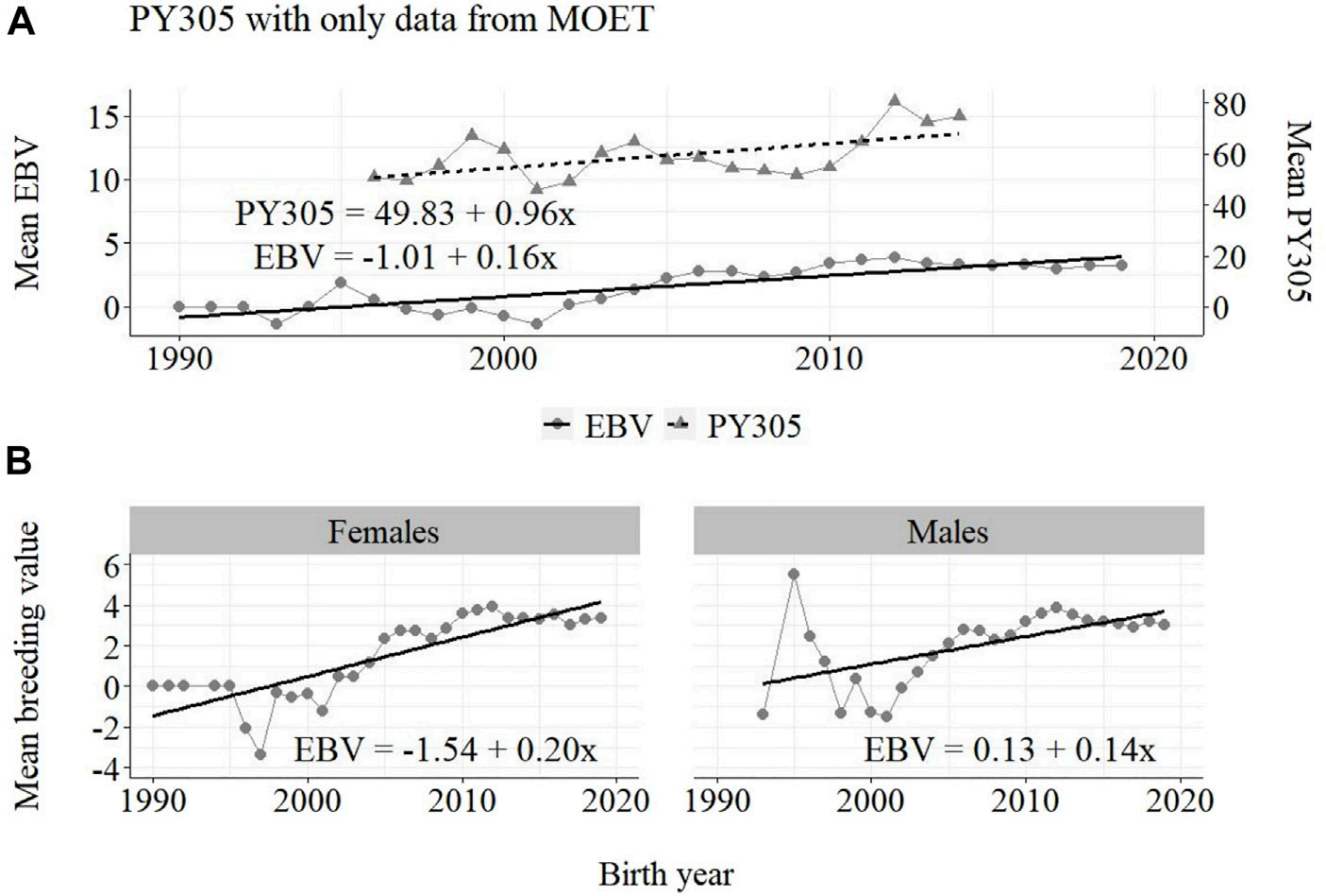
Annual phenotypic and genetic trends for FY305 of Guzerá animals for **(A)** all animals, and genetic trends for **(B)** males and females, considering only data from the MOET nucleus scheme.

The annual phenotypic increases for milk yield reveal favorable improvements of 41 ± 11 kg/year (Figure 1), 17 ± 7 kg/year (Figure 2), and 98 ± 13 kg/year (Figure 3) (*p*-values < 0.001), respectively, for the whole population, the reduced population, and the MOET nucleus. The genetic increases for milk production for the whole population (5.27 ± 0.30 kg/year; *p*-value < 0.001), and the population excluding MOET records (1.05 ± 0.22 kg/year; *p*-value < 0.02), were notably lower than the genetic rate for the MOET Nucleus population (9.39 ± 0.79 kg/year; *p*-value<0.001). The increases in the female and male pathways for the whole population were similar. However, for the MOET scheme, the female pathway contributed more for genetic progress than did the male pathway. Excluding data from the MOET nucleus, though, the male pathway contributed slightly more to the rate of genetic progress.

The annual genetic progress for fat production in the whole population (100 ± 10 g/year; *p*-value < 0.001) and in the population excluding MOET records (5 ± 6 g/year; *p*-value < 0.45; not significant at 5% level) were notably lower than the genetic rate in the MOET Nucleus population (220 ± 28 g/year; *p*-value < 0.001). The female and male pathways behaved the same as for milk production, with the female pathway higher than the male pathway in the MOET nucleus population.

The annual genetic progress for protein production in the whole population (70 ± 7 g/year; *p*-value < 0.001) and in the population excluding MOET records (−10 ± 5 g/year; *p*-value < 0.30; not significant at 5% level) were notably lower than the genetic rate in the MOET Nucleus population (160 ± 20 g/year; *p*-value < 0.001) and, in some cases, were even negative. The genetic progress for protein in the population excluding MOET records can be considered null. The female and male pathways behaved the same as for milk production, with the female pathway higher (36%) than the male pathway in the MOET nucleus population.

**FIGURE 10:**
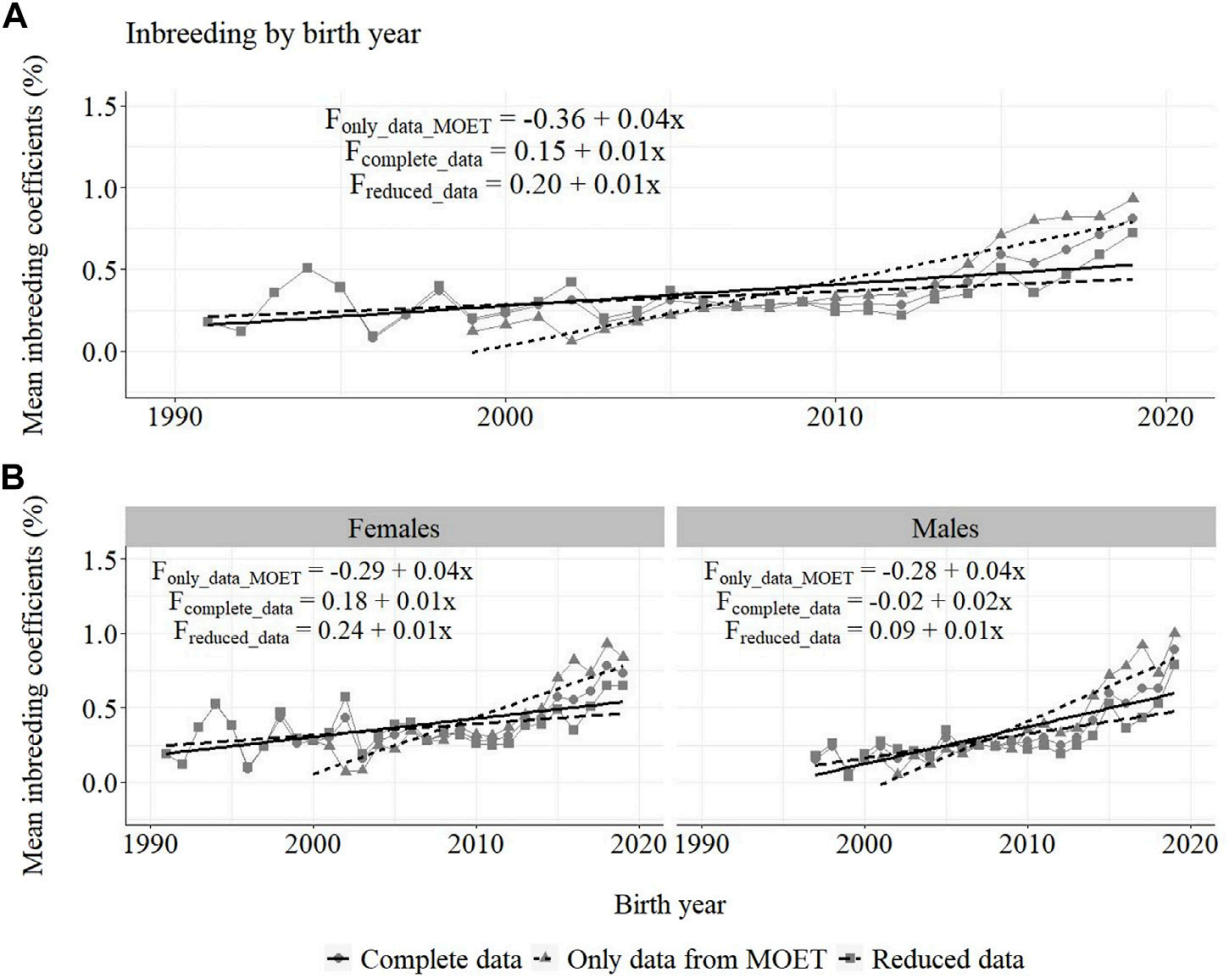
Annual inbreeding trends for Guzerá animals from herds selected for milk purpose, considering data of only MOET animals (F_only_data_MOET_), complete data (F_complete_data_), reduced data (F_reduced_data_) for **(A)** all animals, and for **(B)** male and female pathways.

Annual inbreeding trends for selected Guzerá animals, considering data of only MOET animals (F_only_MOET_), with MOET animals (F_with_MOET_) and without MOET animals (F_without_MOET_) for (A) all animals, and for (B) males and females, can be seen in Figure 10. The increase in the inbreeding coefficient for the MOET scheme (4%) was larger than for the whole population (1%), and was similar between the female and male pathways, except for the male pathway (2%) when F was calculated using the reduced data.

**FIGURE 11:**
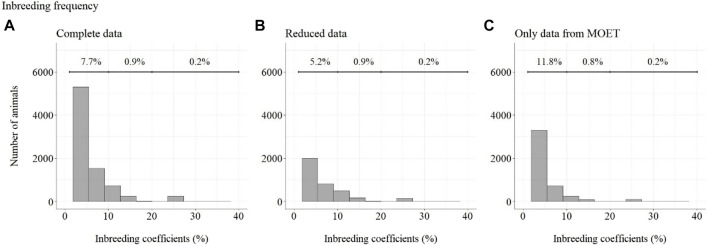
Inbreeding coefficient frequency for selected Guzerá animals, considering **(A)** complete data, **(B)** reduced data, and **(C)** data with only MOET animals. The portion of animals with inbreeding equal to zero was not included.

The frequency of Fs can be seen in Figure 11. The top line in Figure 11 shows the percentage of animals included in each range (F from 1 to 10%, 10–20%, and 20–50%). For ease of viewing, animals with inbreeding equal to zero have not been included in this figure.

Considering complete data, 91.2% of animals presented F equal to zero (not shown), 7.7% presented an inbreeding coefficient less than 10%, 0.9% of animals presented F between 10 and 20%, and 0.2% presented an inbreeding coefficient greater than 20%. Considering reduced data, 93.7% of animals presented F equal to zero (not shown), 5.2% presented F less than 10%, 0.9% of animals presented F between 10 and 20%, and 0.2% presented F greater than 20%. Considering data containing only MOET animals, 87.2% of animals presented an inbreeding coefficient equal to zero (not shown), 11.8% presented an inbreeding coefficient less than 10%, 0.8% of animals presented an inbreeding coefficient between 10 and 20%, and 0.2% presented an inbreeding coefficient greater than 20%.

## 4 Discussion

Although there are many theoretical studies, to our knowledge, there are no practical results on the performance of any other selection nucleus in the literature. This is, therefore, the first study to present such results.

As shown in Figures 1–3, the annual phenotypic progress for milk yield was higher in the MOET nucleus (≅+ 98 kg) than in the population without MOET data (≅+ 17 kg), making evident the success of this scheme. The phenotypic progress for milk yield can also be attributed to some environmental changes that had happened during the selection process, mainly in nutritional and sanitary management, contributing favorably to the increase in milk production in the Guzerá. The annual genetic rate in the MOET Nucleus was 9.39 kg, which is 43% above the genetic progress (+5.27 kg) for the whole population, including data for the MOET animals. Furthermore, it is 80% above the genetic progress (+1.05 kg) for the reduced population, with the removal of data for the MOET animals. However, it is below the theoretical genetic gain (1%–2%) expected, considering some assumptions (Rendel and Robertson, 1950), which could have resulted in a gain between 20 and 40 kg milk/year for the Guzerá cattle. This result reveals the potential for genetic progress in the Guzerá cattle, mainly considering the heritability described for MY305 (0.32 ± 0.04) for this breed (Teepker and Smith, 1990; Carrara et al., 2022). Otherwise, the time to reach the asymptotic rates of genetic gain will be slow (Dentine and McDaniel, 1987). The progress reached in the MOET nucleus also reflects the effects of the favorable environmental conditions in which the MOET animals were raised.

Results from Peixoto et al. (2006), using a similar but smaller database, showed lower, although still positive, genetic progress for milk yield. This was promoted by the PNMGuL 10 years after the beginning of the breeding program and 4 years after the publication of the first sire summary. However, this earlier study found annual genetic progress 6 kg and 10 kg less than the present study for the whole population and for the MOET nucleus, respectively. At that time, this was attributed mainly to the initial genetic lift. As observed in the present study, the genetic progress for milk production in the MOET nucleus was superior throughout the selection process, and was mainly responsible for the genetic progress of the whole population.

Studies conducted in developed countries for other breeds, just to access the genetic possibilities for improvement, have found the highest genetic progress for the milk purpose to be due to directional selection. For the Holstein population in the United States, e.g., higher annual phenotypic (≅+273 kg) and genetic (≅+168 kg) progress in milk production was observed from 1957 to 2020. A continuous and intense selection process focused on milk traits combined with the constant adoption of modern methodologies during this period support this result (CDCB, 2022). For Guzerá cattle in Brazil, on the other hand, herds selected for milk purposes were also selected for beef purposes since the Guzerá is considered a dual-purpose breed. Many herds have a possibility for double-income growth and/or finishing of Guzerá males (Peixoto et al., 2021) in this selection goal and production system strategy. Rangel et al. (2018) found annual phenotypic (+89 kg) and genetic (+27 kg) trends for Gir-breed herds, another *Bos indicus* breed, in a low-input and pasture-based production system and under direct selection for milk production in Brazil from 1987 to 2012. This study, although conducted for only one herd, revealed higher genetic and lower phenotypic progress rates as those obtained for the Guzerá breed, and had the same low selection response. However, to our knowledge, there is no recent study about Gir cattle trends for milk traits. As this breed has long been selected for milk yield, these results probably do not reflect the actual phenotypic and genetic progress for the whole population of this breed.

Furthermore, in an attempt to control inbreeding, the influence that the strategy of introducing animals from different lineages or lineages still not used, as well as risk animals about which little was known could have contributed to the timid results for genetic gain for milk production. Joint to the mating planning implemented since the beginning of the nucleus scheme, inbreeding has been an important and constant concern.

The results for the fat yield revealed low or even negative annual phenotypic progress, despite the positive genetic progress in the population, both with (−1 kg and +100 g, respectively) and without (−1 kg and +5 g, respectively) data from the MOET nucleus. For this trait, the progress in the MOET scheme had increased phenotypically and genetically by 380 g and 220 g of fat/year, respectively. It seems that these increases could not have contributed in any meaningful way to the improvement of this trait in the population. Two aspects should be taken into account. First, the genetic correlation between milk and fat yield (0.96 ± 0.02) in Guzerá (Carrara et al., 2022), although high, is not perfect and the genes’ action on these traits may be different. Thus, no proportional genetic progress in fat yield, due to direct selection for milk production, is expected (Meinert et al., 1988, Quinton and MacMillan, 1996, van Binsbergen et al., 2012). Second, in the population as well as in the MOET nucleus, the selection goal has focused on the improvement of milk yield. Thus, the indirect selection response could not have changed the Guzerá fat production in the same proportion despite the high genetic correlation between milk and fat production. Environmental, mainly nutritional (Shingfield et al., 2013), aspects could also have influenced the results for the phenotypic progress of the present study, not allowing the expression of the genetic potential.

For the protein yield, the phenotypic (+320 g) and genetic (+70 g) progress was positive in the whole population. Considering the negative annual phenotypic (−160 g) and genetic (−10 g) progress for the population without data for the MOET animals, and considering the notable annual phenotypic (+960 g) and genetic (+160 g) progress in the MOET nucleus, the progress in the whole Guzerá population can be also attributed to the contribution of the indirect selection for protein production in this scheme. This was because the genetic correlation between milk and protein production (0.97) is high (Carrara et al., 2022). Therefore, the results found for fat and protein production should have occurred as a correlated response to selection since the selection in the Guzerá MOET nucleus had focused on milk production.

Unfortunately, other traits important for dairy systems, such as those related to reproduction, growth and development, morphology, and disease occurrence, were not recorded in the MOET nucleus due to financial and logistic circumstances what would have allowed to evaluate the impact on other important traits. Information on the beef traits of the Guzerá breed, for instance, is processed in specific genetic evaluations for beef cattle, carried out by beef improvement programs. However, as the database is genetically connected to the database of the milk program, the joint availability of genetic values for dual-purpose bulls has been allowed (Bruneli et al., 2021). There are concerns with the necessity of not only enlarging the database for milk and beef traits, but also with implementing the new-traits phenotyping. These concerns arise in view of demands imposed recently, and demands expected to be imposed in the future, on the livestock (Brito et al., 2021).

It is important to clarify in this publication that Guzerá breeders who opted for the dual-purpose systems have no interest in specializing their herds, so the model of maximizing genetic gains in specific traits is not their objective.

The genetic progress achieved for milk yield, from the selection of female and male pathways in the whole population, was similar. However, in the MOET nucleus, the genetic progress from the female pathway (+11 kg) contributed more than that from male pathway (+6.5 kg) to the genetic progress in this scheme. Excluding data of the MOET nucleus from the whole population, the male pathway contributed slightly more (+1.31 kg) to the rate of genetic progress, although it was quite similar to the female pathway contribution (+1.15 kg). Probably, progeny from test-proven bulls also contributed to this slight difference. In a study with the Gir breed in Brazil, Balieiro et al. (2000) found positive, higher phenotypic and genetic progress in both pathways. The Gir genetic progress was higher in the male pathway, which was the main contribution responsible for the improvement of milk traits in the population. The breeding program with the Gir breed, however, was initiated in 1985, 10 years before the PNMGuL, and since then has focused only on milk production traits.

Observing the genetic progress of Holstein or Red and White female and male pathways in USA, it can be noticed that genetic progress came mainly from the progeny of test-proven bulls and recently from the inclusion of genomic data in genetic evaluations, although the contribution of the females is also high and follows the same trend (CDCB, 2022). To our knowledge, there are no current results published for the Gir breed, but the progress in taurine breeds reinforces the potential of progeny-tested bulls as well as the genomic selection to improve milk traits. The MOET nucleus was closed in 2018 and progeny test-based selection should be, henceforth, the main genetic scheme to promote phenotypic and genetic changes in the Guzerá population. It will require both the enlargement of the phenotypic and genomic database for the prediction of accurate breeding values, as well as a target selection program, which is a priority for the PNMGuL at this time. A genomic database is being established for further studies and to conduct more accurate genetic evaluations. Genomics could improve the selection of Guzerá cattle, increasing the genetic gain for the traits of interest (Meuwissen et al., 2001; Pryce and Daetwyler, 2012; Bouquet et al., 2015).

From Figures 4–6, it can be noticed that the genetic progress for fat production in each selection pathway was positive. These results reveal the similarity between genetic progress from selection in the female (+100 g) and male (+110 g) pathways in the whole population. The low and similar genetic progress from female (+10 g) and male (+20 g) pathways also highlights the contribution of the MOET nucleus to the genetic improvement of protein yield, mainly from the female pathway (+250 g), even though this trait was not directly selected. The male pathway contributed slightly less (+200 g). The female and male trends for protein production in the whole population were equal (+70 g), as shown in Figure 7. The main contribution for milk production came from the female pathway (+200 g), rather than from the male pathway (+140 g). The removal of data from this scheme revealed the negative and low genetic progress from the female (−3 g) and male (−0 g) pathways.

It is important to consider that the inclusion of risk animal/lineage options in the PT and in the MOET nucleus, which was used as an attempt to minimize the inbreeding coefficient progress in the population, also contributed to the low phenotypic and genetic progress found in the present study. However, it was an important approach, which together with the mating planning carried out in the population as well as in the MOET nucleus scheme allowed control for the increase of F in the first years, thus maintaining this parameter at acceptable levels in the population. Concern about inbreeding led the PNMGuL to continuous monitoring of the genetic contribution of bulls by calculating annually the average relationship coefficient and making available proven bulls of different origins. Breeders are guided breeders to diversify the use of bulls in their herds. The Optimum Contribution Selection is an important strategy to avoid unbalanced use of bulls and decrease the rate of inbreeding (Sorensen and Sorensen, 2009).

It is also important to stress that the Guzerá breed went through many bottlenecks during its evolution in Brazil, mainly as a result of its intense use in crossbreeding and the formation of synthetic breeds (FAO, 1998; Fonseca et al., 2016). Therefore, the small effective size of the population requires caution and continuous monitoring. Another aspect to be considered is related to the discarding of highly-inbred animals during this period, probably due to low reproductive traits. This aspect can imply biased estimates of the inbreeding coefficient.

In 2000, the first Guzerá sire summary containing the results of accurate genetic evaluations was made available and producers felt confident in using bulls from other origins, which also contributed to keeping low F levels. The positive impact of the breeding program on the F of the population became evident from 2003 to 2012, when the progeny of proven sires were born. After this period, it started to increase again, maybe due to the use of superior bulls from the same family that will require new control efforts. The intense use of MOET proven bulls, the top of the sire-summary ranking, probably contributed to the increase of F in the population after 2012, as shown in Figure 11. Otherwise, it should be emphasized that the control for F in the population, despite being rated below the MOET nucleus, stopped working. With awareness of the risk of increasing inbreeding in the Guzerá population (Fonseca et al., 2016; Peixoto et al., 2021), studies were carried out on population structure and genetic diversity in order to understand the gene flow in the population and identify strains that could be used strategically in planning the mating of herds.

In an interesting review, Brito et al. (2021) presented the huge phenotypic and genetic progress obtained worldwide with selection for dairy cattle at the expense of adaptive traits. This trade-off has resulted in important losses in reproductive and health traits, mainly as a consequence of inbreeding depression. This is an important concern in any breeding program since adaptive traits are closely related to production and reproduction efficiency, as well to revenue, which could impair the sustainability of the dairy production system. Therefore, since the beginning, the PNMGuL has enlarged its genetic basis using the MOET nucleus scheme for previously testing risk options and new lineages.

## 5 Conclusion

In conclusion, we can say that the MOET Nucleus scheme has allowed the genetic improvement of Guzerá cattle, genetically lifting the dual-purpose population for milk production in realistic, tropical production systems, and contributing to the economic return of the activity. The low phenotypic progress of the Guzerá population is partially due to the low-medium input production systems. These production systems impair the expression of the genetic potential and limit the possible phenotypic trends, as well as raise concerns about losses of genetic variability due to the increasing inbreeding coefficient. The selection for milk production in MOET nucleus allowed correlated response in fat and protein production, albeit small. The PNMGuL should start phenotyping for other important traits as soon as possible, in view of the prospects of future demands, focusing on the sustainability of the program. The increase in F in recent years will demand an implementation of a mating strategy to minimize the consequences of family selection based on the scheme of PT bulls. The MOET nucleus scheme represents an important tool for the rapid and easy change of production traits, requiring a well-organized partnership.

## Data Availability

The data analyzed in this study is subject to the following licenses/restrictions: Dataset belongs to the National Breeding for the Improvement of Guzerá. This data is being used for many genetic studies. So, this availability is possible only through a direct demand to the Brazilian Center for the Genetic Improvement of Guzerá (CBMG2) and authorization is possible through a NDA term. Requests to access these datasets should be directed to frank.bruneli@embrapa.br.
